# Deep learning forecasting using time-varying parameters of the SIRD model for Covid-19

**DOI:** 10.1038/s41598-022-06992-0

**Published:** 2022-02-22

**Authors:** Arthur Bousquet, William H. Conrad, Said Omer Sadat, Nelli Vardanyan, Youngjoon Hong

**Affiliations:** 1 Department of Mathematics and Data Science, Lake Forest College, Lake Forest, CA USA; 2 Department of Chemistry, Lake Forest College, Lake Forest, CA USA; 3 Department Mathematics, Sungkyunkwan University, Suwon, South Korea

**Keywords:** Computational models, Machine learning, Computational science, Information technology

## Abstract

Accurate epidemiological models are necessary for governments, organizations, and individuals to respond appropriately to the ongoing novel coronavirus pandemic. One informative metric epidemiological models provide is the basic reproduction number ($R_0$), which can describe if the infected population is growing ($R_0 > 1$) or shrinking ($R_0 < 1$). We introduce a novel algorithm that incorporates the susceptible-infected-recovered-dead model (SIRD model) with the long short-term memory (LSTM) neural network that allows for real-time forecasting and time-dependent parameter estimates, including the contact rate, $\beta$, and deceased rate, $\mu$. With an accurate prediction of $\beta$ and $\mu$, we can directly derive $R_0$, and find a numerical solution of compartmental models, such as the SIR-type models. Incorporating the epidemiological model dynamics of the SIRD model into the LSTM network, the new algorithm improves forecasting accuracy. Furthermore, we utilize mobility data from cellphones and positive test rate in our prediction model, and we also present a vaccination model. Leveraging mobility and vaccination schedule is important for capturing behavioral changes by individuals in response to the pandemic as well as policymakers.

## Introduction

According to the World Health Organization (WHO), over 5 million people worldwide have died from Covid-19^1^. Public health interventions have limited incidence and mortality of this disease from an early stage^2^. Governments, public health institutions, and the public at large benefit from statistical models that help to determine what approaches are effective at controlling the virus, and to predict when it is necessary to take strong measures to slow its transmission. For instance, recent studies have shown the benefits of both voluntary and government-induced social distancing measures^3^. A key metric to predict epidemic progression is the basic reproduction number ($R_0$)^4^. Both compartmental models and networked models have been used to predict $R_0$^5–14^. Compartmental models such as the SIRD (susceptible, infectious, recovered, or dead) model and its variants are used to predict $R_0$ for infectious diseases^15,16^, because the number of susceptible, infectious, recovered, and dead people in a population can be readily estimated from publicly available data. The epidemiological parameters $\beta$ (contact rate), $\gamma$ (recovery rate), and $\mu$ (deceased rate) in the SIRD model can be esteimated from the number of the susceptible, infectious, recovered, and dead. These parameters are then used to determine $R_0$.

The SIRD model (see Eq. 2) can be solved in different ways. One of the popular methods is to solve the ordinary differential equations (ODEs) using numerical methods. In this case, one needs to know the parameters ($\beta$, $\gamma$, and $\mu$) of the system of differential equations. However, if these parameters are set to be a time-independent constant, the assumption may not be realistic. For example, the contact rate, $\beta$, varies depending on many time-dependent factors such as mobility and lockdown policy. Hence, time-independent parameters may give outdated information, which introduces a prediction error. Another method to solve the equations is to use neural networks by considering the system as a time series^17^ with a recurrent neural network^18^. This approach does not ensure that the model follows the dynamics of compartmental models, and the neural network is required to predict twice as many variables. More importantly, this approach does not provide the reproduction rate $R_0$ directly.

Recently, related studies about Covid-19 incorporated mobility datasets to aid in pandemic modeling^19–21^. For example, James and Menzies^19^ used Apple mobility data to examine the relationship between daily Covid-19 cases and national equity index price on a country-by-country basis. Yilmazkuday^20^ studied the relationship between country-specific changes in mobility, from the Google mobility dataset, and the number of Covid-19 cases. Also, a metapopulation SEIR model was investigated in^21^ that integrated fine-grained dynamic mobility from Safegraph data to simulate the spread of Covid-19. Each of these studies demonstrated that by integrating these mobility data, the SEIR model can accurately fit the real case trajectory, despite substantial changes in the behavior of the population over time.

In this work, we combine a compartmental model with a recurrent neural network that incorporates mobility data as well as the positive test rate. We (1) predict the time-dependent parameters $\beta$ and $\mu$ using a neural network; (2) forecast the infection rates when mobility decreases or increases; and (3) forecast the change in infection rate based on different vaccination schedules. The goal of this paper is to provide a method to predict time-varying parameters $\beta$ and $\mu$ (and hence $R_0$) as well as to solve the SIRD equations.

The method under consideration in our paper combines the two aforementioned approaches. We first introduce a version of recurrent neural networks to predict the time-varying parameters $\beta$ and $\mu$. Since $\gamma$ is assumed to be constant, one can easily find $R_0=\beta /\gamma$ from the neural network. We then obtain the compartments, S, I, R, and D, by numerically solving the SIRD equation over a certain time period (e.g. 7 days). To test the performance of our approach, we used publicly available data for different countries, France, United Kingdom, Germany, and South Korea, provided by Johns Hopkins University. For more detail, we provide an illustration of the algorithm in Fig. 9. We also include two additional datasets: mobility data from cellphones and the positive test rate. Studies reveal that both mobility and positive test rate have been shown to influence the spread of Covid-19 considerably^22–25^.

In this paper, we present an accurate computational scheme to predict the reproduction number which enables Covid-19 forecasting. We use this scheme to forecast different scenarios by increasing or decreasing the mobility parameter. In doing so, our model can help study the effect of government-imposed lockdowns on $R_0$. Furthermore, we make use of a SIRD model with vaccination to see how vaccination affects the spread of the virus. Among many other vaccination models^26,27^, our study focuses on the model introduced in^28,29^ as it is sufficient to capture important dynamics in the experiments. By leveraging parameters relative to the vaccination rates, our simulations show how the vaccination rate affects the number of infectious cases. Such experiments can show how different public health interventions may affect the outcome of the epidemic.

## Results

In this section, we describe a sequence of numerical experiments of our algorithm further detailed in the Method section below. First, we present the estimated values of our time-dependent parameters $\beta$ and $\mu$ using the Levenber–Marquardt algorithm. Then, the accuracy of the algorithm is demonstrated using in-sample data, and out-of-sample predictions for the next 10 weeks. Lastly, forecasting depending on mobility and vaccination rate is examined. In summary, our main contributions consist of three key findings; (i) our SIRD–LSTM combined network outperforms classical prediction models; (ii) we incorporate the mobility and vaccination as inputs of our neural network to increase the accuracy of our parameters predictions; (iii) we forecast Covid-19 trends when mobility decreases or increases.

### Parameter Estimates

**Figure 1:**
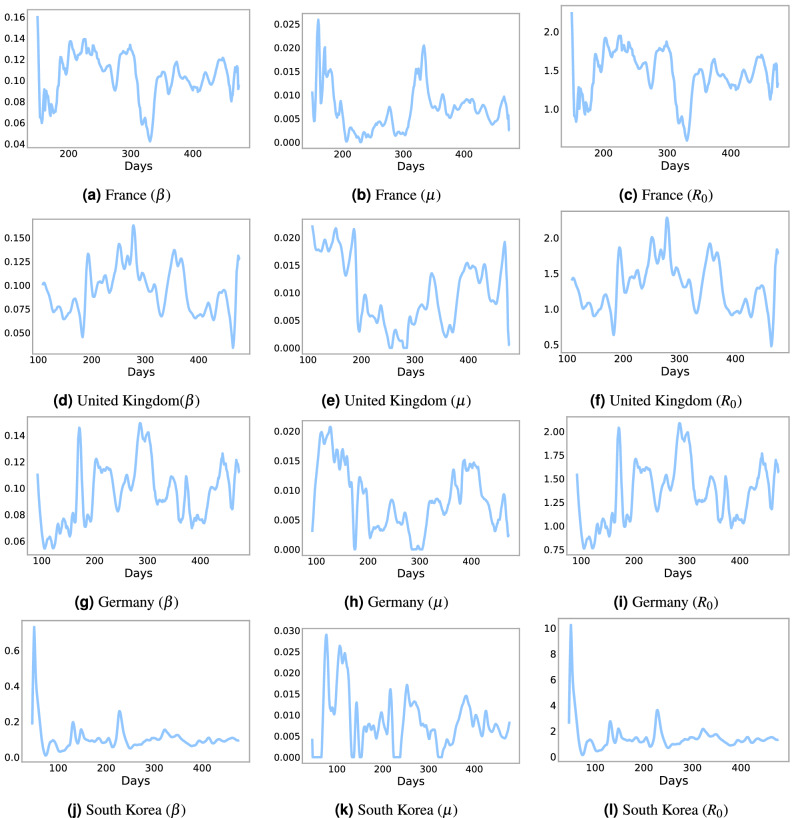
Predicted time varying parameters $\beta$ (contact rate), $\mu$ (deceased rate), and $R_0$ reproduction number for each country derived from the Levenberg–Marquardt algorithm.

**Figure 2:**
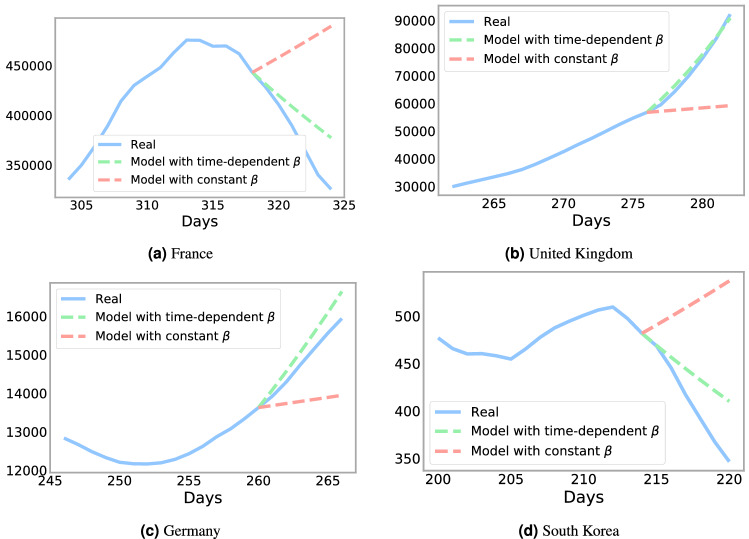
Comparison between the number of infected, *I*, from our data with a predicted *I* using the SIRD model using a constant $\beta$ or a time-dependent $\beta$.

A significant finding of our paper is that treating the parameters $\beta$ and $\mu$ as time-dependent increases model accuracy. Figure 1 shows $(\beta ,\mu )$ for four countries (France, United Kingdom, Germany, and South Korea) generated by the Levenberg–Marquardt algorithm. From this, we can find the basic reproduction number, $R_0 = \frac{\beta }{\gamma }$, with $\gamma = 1/14$, which is useful to study the dynamics of the infectious class^30^. We compare real infection data from France, the United Kingdom, Germany, and South Korea with a SIRD model using constant $\beta$ or time-dependent $\beta$. Figure 2 shows the difference between a $\beta$ and $\mu$ constant, that we estimate using the Levemberg–Marquardt algorithm over one year, with $\beta$ and $\mu$ estimated over just 1 week. The time-dependent model more accurately forecasts the infection rate over seven days across each country regardless of the time period. Therefore, it is necessary to consider $\beta$ and $\mu$ as time-dependent variables.

### Accuracy of our model

**Figure 3:**
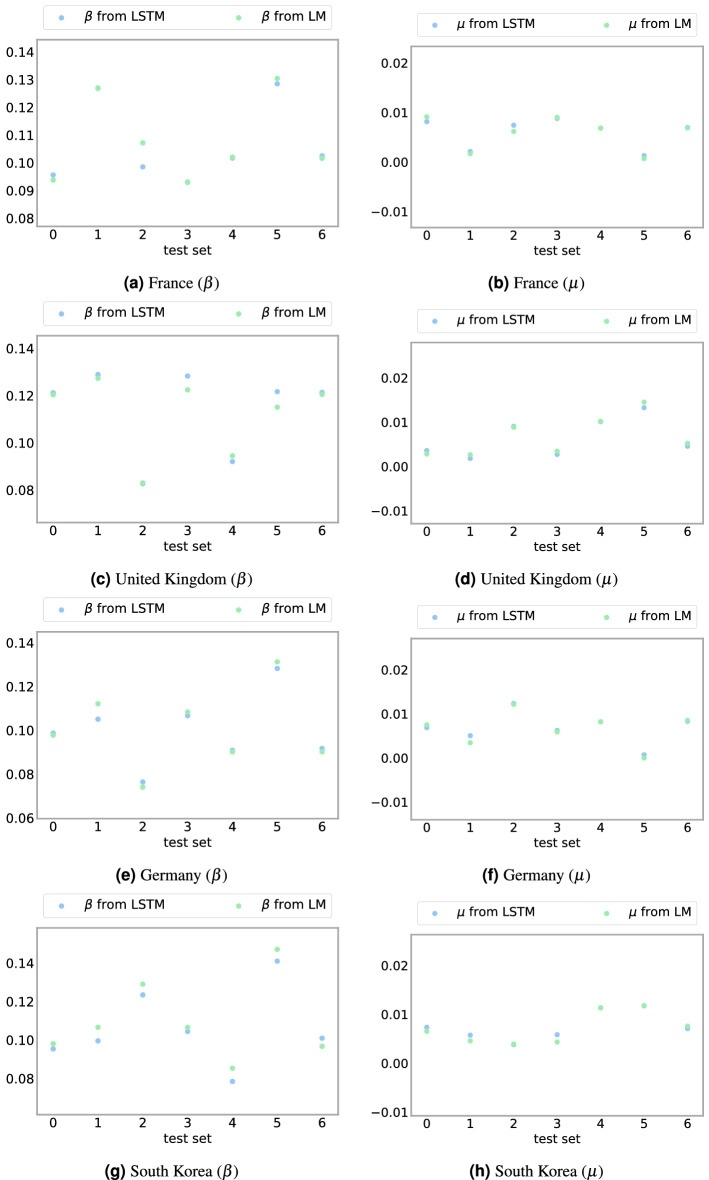
Comparison between $(\beta ,\mu )$ computed by the Levenberg–Marquardt (LM) algorithm which is considered as our true data, and ($\beta$, $\mu$) predicted from the LSTM networks for France, United Kingdom, Germany, and South Korea.

To test the forecasting capability of the SIRD–LSTM combined network, we compare the number of predicted confirmed Covid-19 cases under various measures for within sample scenarios. The in-sample fit of the model is an essential indicator for the validity of the model’s prediction of the parameters, whereas the out-of-sample forecasts can provide an important guideline for decision/policymakers. Figure 3 depicts the prediction of the time varying parameters $(\beta ,\mu )$ compared with $(\beta ,\mu )$ from the dataset. We randomly choose $N_T$ test data amongst 365 days, and make use of them as a test set. To measure the accuracy, we use the relative-$L^2$ errors of $\beta$, $\mu$, *S*, *I*, *R*, and *D* such that

1 $$\begin{aligned} {\text {Errors} = \frac{1}{N_T} \sum _{i=1}^{N_T} \frac{\Vert Y_{true}^i - Y_{pred}^i \Vert _{L^2}}{\Vert Y_{true}^i \Vert _{L^2}}, \; \quad N_T = \text {number of test days (test set)},} \end{aligned}$$

where $Y_{true}^{i}$ is the ith true dataset of $\beta$, $\mu$, *S*, *I*, *R*, or *D*, and $Y_{pred}^{i}$ is *i*th predicted values from our algorithm. We observe that the predicted and true parameters are close to each other. Table 1 demonstrates quantitative results on accuracy of our computation.

**Figure 4:**
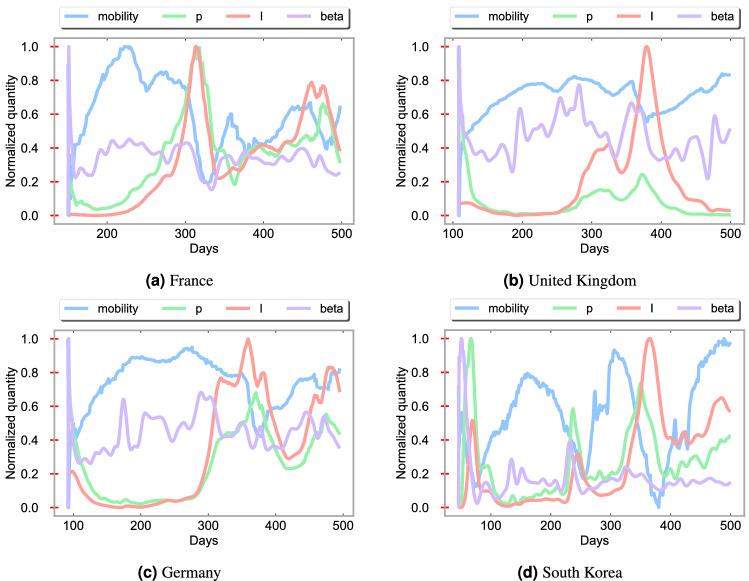
Plots of the normalized mobility values, positive test rate (p), infectious individual (I), and contact ratio ($\beta$) against the time (Days) for France, United Kingdom, Germany, and South Korea.

Table 1 shows the relative $L^2$ error of $\beta$ is between $3.13\times 10^{-3}$ and $6.29\times 10^{-2}$, the relative $L^2$ error of $\mu$ is between $9.26\times 10^{-2}$ and $1.73\times 10^{-1}$. The relative $L^2$ error with $N_T=14$ (2 weeks), of the compartments, *S*, *I*, *R*, and *D*, is also displayed in Table 1. Figure 4 depicts mobility, positive test rate, cumulative infectious individuals, and contact ratio $\beta$ against the time. The positive test rate and cumulative infectious individuals follow similar trends as opposed to mobility and the positive test rate. The countries under consideration enforce lockdowns as cumulative infectious individuals increased. Hence, the trend plots reveal that greater mobility leads to an increase in infectious individuals.

**Table 1:** Relative $L^2$ errors with $N_T=14$ (2 weeks) for $\beta$ and $\mu$, and for the SIRD implementation with the LSTM networks.

Country	$\beta$	$\mu$	*S*	*I*	*R*	*D*
France	$3.17 \times 10^{-3}$	$1.02 \times 10^{-1}$	$9.4 \times 10^{-5}$	$4.2 \times 10^{-2}$	$6.3 \times 10^{-3}$	$6.3 \times 10^{-2}$
United Kingdom	$3.13 \times 10^{-3}$	$9.26 \times 10^{-2}$	$1.7 \times 10^{-4}$	$3.8 \times 10^{-2}$	$1.8 \times 10^{-3}$	$2.2 \times 10^{-2}$
Germany	$3.11 \times 10^{-3}$	$9.71 \times 10^{-2}$	$5.4 \times 10^{-5}$	$2.1 \times 10^{-2}$	$1.1 \times 10^{-2}$	$1.3 \times 10^{-1}$
Korea	$6.29 \times 10^{-2}$	$1.73 \times 10^{-1}$	$8.0 \times 10^{-7}$	$9.4 \times 10^{-3}$	$2.0 \times 10^{-3}$	$3.4 \times 10^{-2}$

SIRD stands for susceptible (S), infectious (I), removed (R), deceased (D) individuals. The definition of relative $L^2$ error is stated in (1).

### Out-of-sample forecast

**Figure 5:**
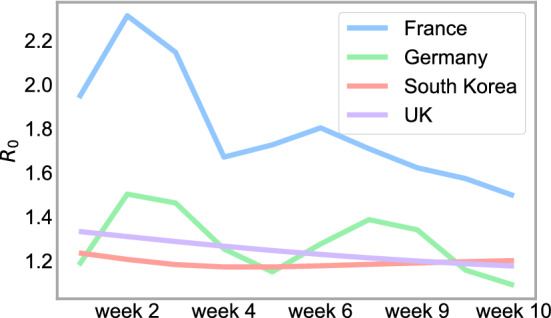
10 weeks prediction of the reproduction number, $R_0$, using $\beta$ generated by the LSTM network for each country.

**Figure 6:**
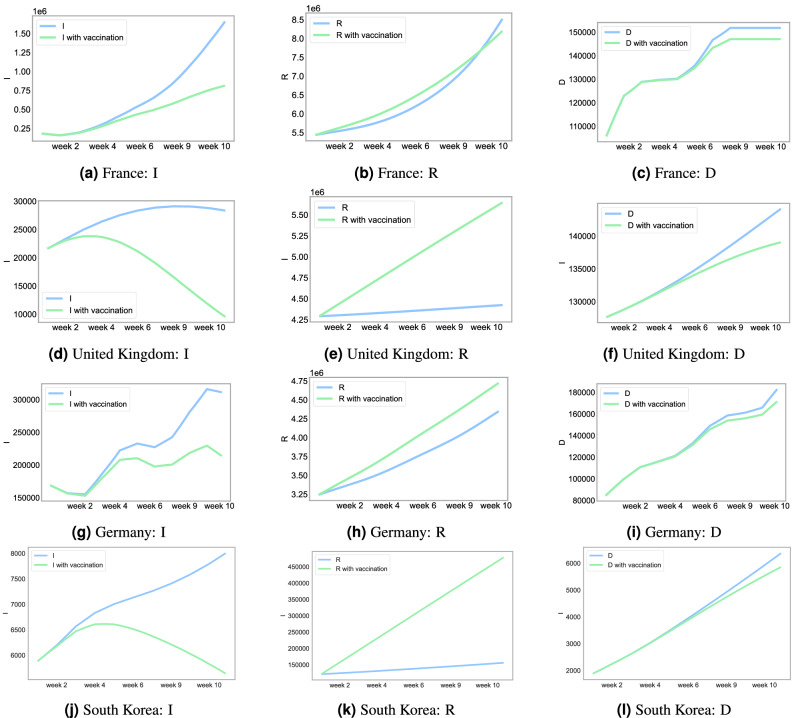
The SIRD prediction with the LSTM network for France, United Kingdom, Germany, and South Korea. The plots display I, R, and D prediction against the time (Days) for 10 weeks.

We next conduct an out-of-sample forecast analysis of our SIRD–LSTM combined model. Figure 5 demonstrates a prediction of $R_0$ of each country using $\beta$ generated by the LSTM networks. By forecasting $\beta$, in Fig. 6, we show a short-term prediction of the SIRD model up to 10 weeks. In the simulation, we assume that the positive test rate and mobility are the same as the final observation from the dataset. Both the SIRD and vaccinated SIRD models are computed and demonstrated in Fig. 6. In France, Germany, and South Korea, the depicted curves of the infections for the next 10 weeks are increasing, while the infection curve for the next 10 weeks tends to slightly decrease in the United Kingdom. In fact, it has been reported from various sources in May 2021 that the vaccination strategy and lockdowns in the United Kingdom were successful^31^.

### Forecasting depending on mobility

**Figure 7:**
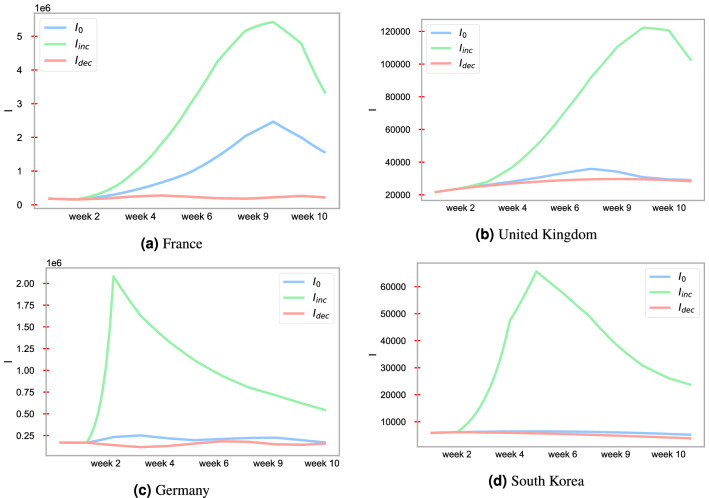
Forecasting the number of Covid-19 infections for France, the United Kingdom, Germany, and South Korea under 30% increased and decreased mobility to normal mobility (baseline mobility). Mobility data is real-time cell phone/mobile device location for each country collected from^32^. Here, $I_0$, $I_{inc}$, and $I_{dec}$ stand for infections with normal, 30% increased, 30% decreased mobility, respectively. The vaccination model is used for the simulations.

Policymakers have sought to decrease the rate of infection in their populations by decreasing population mobility through lockdowns and, more recently, increasing vaccinations. Here, we model the effect of decreasing mobility and increasing vaccination rate on infection rate. If the mobility is increased by $30\%$ of the normal mobility (baseline mobility), the model shows that the peak of infectious individuals increases drastically, see Fig. 7. The data show how visits to places are changing compared to the baseline. A baseline day represents a normal value for that day of the week. The baseline day is the median value from the 5 weeks Jan 3–Feb 6, 2020; for more information, see e.g.^32^. Figure 7 shows that in France, South Korea, and Germany, increased mobility results in a drastic change in the number of new Covid-19 cases. On the other hand, if mobility restrictions are decreased to 30% normal mobility, the model predicts that the peak of infectious individuals decreases compared to the baseline mobility.

### Forecasting depending on the vaccination rate

**Figure 8:**
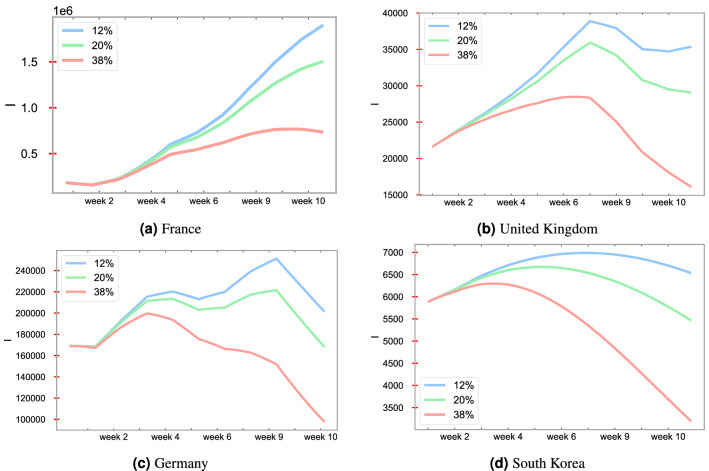
Forecasting of of the number of Covid-19 infections for France, the United Kingdom, Germany, and South Korea under various vaccination schedules. Here, “$12\%$", “$20\%$", and “$38\%$" mean $12\%, 20\%, 38\%$ of the population is vaccinated, respectively.

In addition, with vaccination, the Covid-19 cases are noticeably decreasing for all of the countries under study in our work. The countries whose reproduction number ($R_0 = \beta /\gamma$) is close to 1 such as the United Kingdom and South Korea, have a better vaccination effect than the other countries. Figure 8 displays forecasting of infectious cases under various vaccination schedules within 70 days. In the experiment, we assume that the vaccine is evenly distributed with respect to time. The plots reveal that high vaccination rates are important in reducing the number of infectious cases. Figure 7 shows the models’ forecast for infections with different mobility levels in each country. Given mobility information, the combined SIRD–LSTM model can predict the time-varying parameters $(\beta ,\mu )$. With those predicted parameters, the number of infectious individuals are implemented with or without vaccination. Based on the projected forecasts, we observe that a continuation of quarantine level mobility will result in low case counts.

## Discussion

We introduced a novel algorithm that incorporates deep learning and compartmental models allowing for forecasts and evaluation of the current Covid-19 outbreak worldwide. We combined the SIRD model with the LSTM network and observed advantages of real-time forecasting and parameter estimation. The new algorithm integrates the forecasting accuracy of LSTM networks with the epidemiological model dynamics of the SIR-type model. Compared to the classical SIRD model in the literature, we forecast time-varying parameters predicted by the LSTM neural network. To forecast the parameters, mobility and positive test rate data are used in the architecture. We find that these inputs are important in improving the model’s ability to fit the data. In addition, incorporating these data is essential for capturing behavioral changes by individuals in response to the pandemic as well as to observe the effect of policy decisions to increase vaccination and decrease mobility. As in other approaches, we conduct our research on publicly available datasets. We demonstrate how a new algorithm can be developed to better exploit quantitative measures in the fight against Covid-19. By utilizing reliable metrics and infection dynamics, we provide an approach that is deeply data-driven and computer-based. The proposed simulations can provide a tool for forecasting the effects of different mobility scenarios. Furthermore, as the proposed algorithm is compatible and generalizable, this allows for additional compartments in the SIR model or additional input datasets in the network which makes the method accessible to policymakers.

Our developments point towards several extensions of great importance. In particular, we evaluated the impact of the imposition and relaxation of lockdown measures by inputting these changes into the LSTM neural network. We found that employing lockdown rules for each country can help to capture interesting regional dynamics of the Covid-19, and may give specific information to the policymakers. Another direction is to study an advanced deep learning architecture such as attention mechanism or transformer^33^. These modern architectures can provide better investigation on not only the increase in forecasting performance but also on how the highly nonlinear capabilities of the neural network can be used to conduct inference on latent parameters of the SIR model.

## Methodology

In this section, we explore our numerical method and prediction algorithm considered in this research. To begin, we describe the compartmental models, the SIRD equations, and the Runge–Kutta method. Then, we present the Levenberg–Marquardt algorithm. Lastly, we illustrate the combined SIRD–LSTM architecture which is the heart of our approach. We confirm that all methods were performed in accordance with the relevant guidelines and regulations.

### Compartmental model: SIRD model

In this study, we represent the spread of Covid-19 using the susceptible-infected-recovered-dead (SIRD) model. Compartmental models have been used to simplify the mathematical modeling of infectious diseases^34,35^. One of the well-known (and simplest) models is the SIR model, and many models including SIRD are derivatives of this basic form^36–38^. The SIRD model predicts how a disease spreads, the total number infected, or the duration of an epidemic, and estimate important epidemiological parameters such as the reproductive number. Regarding the compartmental model, the population is assigned to compartments with labels:

- *S*(*t*): the number of individuals susceptible of contracting the infection at time *t*,
- *I*(*t*): the number of individuals that are alive and infected at time t;
- *R*(*t*): the cumulative number of individuals that recovered from the disease up to time t;
- *D*(*t*): the cumulative number of individuals that deceased due to the disease, up to time t.

In addition, *N* is the total number of people in the area at time *t* with $N = S(t) + I(t) + R(t)$. The SIRD model is given by the following expressions^15^:

2 $$\begin{aligned} \begin{aligned} \dfrac{dS}{dt}&= -\frac{\beta I S}{N}, \\ \dfrac{dI}{dt}&= \frac{\beta I S}{N} - \gamma I - \mu I,\\ \dfrac{dR}{dt}&= \gamma I,\\ \dfrac{dD}{dt}&= \mu I, \end{aligned} \end{aligned}$$

where the parameter $\beta$, called the contact ratio, represents the effective contact rate, i.e. expected number of people infected by an infectious person, and $\gamma$ is defined as recovery rate, i.e. expected number of people removed from the infected state. The ratio of $\beta$ and $\gamma$ is called as reproduction number, i.e. $R_0$ = $\beta /\gamma$. The reproduction number ($R_0$) shows the average number of secondary infections coming from an infected person. The parameter $\mu$ is defined as a deceased rate. We assume that the recovered subjects are no longer susceptible to infection; the number of deaths due to other reasons is neglected. Further, the region under consideration is assumed to be isolated from other regions. This is a reasonable assumption as containment measures such as travel restriction have been enforced in most countries.

By introducing the vaccination rate,

3 $$\begin{aligned} v = \frac{\text {target population}}{\text {completion time}} \end{aligned}$$

the *S*(*t*) and *R*(*t*) terms can be modified for the vaccination model. We add the vaccination rate, $\nu$, and the vaccine efficacy factor, $\varepsilon$, into our SIRD model to study an extended SIRD model with vaccination. For instance, $\varepsilon =0.95$ for the Moderna and Pfizer vaccine^39^. More precisely, we introduce a multiplier factor $\delta =(1-\varepsilon )$.

We now write the following SIRD model which incorporates vaccination^28,29^

$$\begin{aligned} \dfrac{dS}{dt}&= -v \delta -\frac{\beta I S}{N}, \\ \dfrac{dI}{dt}&= \frac{\beta I S}{N} - \gamma I - \mu I,\\ \dfrac{dR}{dt}&= v(1-\delta ) + \gamma I,\\ \dfrac{dD}{dt}&= \mu I. \end{aligned}$$

With the SIRD model, we generate a deep neural network to predict $\beta$ and $\mu$. Subsequently, the SIRD with vaccination model provides the dynamics of the vaccination with predicted parameters $\beta$ and $\mu$.

The contact rate, $\beta = \beta (t)$, and death rate, $\mu = \mu (t)$, of many acute infectious diseases varies significantly in time and frequently exhibits significant seasonal dependence^40,41^. Epidemiological models can be used to predict contact and death rate, which are important for measuring the spread of disease. A substantial body of research predicts the contact and death rate, $\beta$ and $\mu$, of infectious diseases via the discrete compartmental model^42–44^. The rest of this section introduces an algorithm to compute the time-dependent parameters directly from our data and the discrete SIRD model.

### Levenberg–Marquardt algorithm

To estimate the contact rate, $\beta$, and the death rate, $\mu$, we use the Levenberg–Marquardt algorithm. To apply the algorithm, we solve the SIRD equations using a numerical approximation. In the present study, we use the fourth-order Runge–Kutta methods (RK4) which give the following discrete version of the SIRD model. For simplicity, we set

$$\begin{aligned} \mathbf{y}= \begin{pmatrix} S\\ I\\ R\\ D \end{pmatrix}, \quad \mathbf{F}= \begin{pmatrix} -\dfrac{\beta I S}{N} \\ \dfrac{\beta I S}{N} - \gamma I - \mu I \\ \gamma I \\ \mu I \end{pmatrix}, \end{aligned}$$

then (2) can be recast

4 $$\begin{aligned} \dfrac{d \mathbf{y}}{dt} = \mathbf{F}(t,\mathbf{y}). \end{aligned}$$

The RK4 of (2) can be written as

$$\begin{aligned}&\mathbf{y}_{n+1} = \mathbf{y}_{n} + \dfrac{h}{6} \left( \mathbf{k}_1 + 2 \mathbf{k}_2 + 2 \mathbf{k}_3 + \mathbf{k}_4 \right) ,\\&\mathbf{k}_1 = \mathbf{F}(t_n, \mathbf{y}_n), \quad \mathbf{k}_2 = \mathbf{F}(t_n + h/2, \mathbf{y}_n + h \mathbf{k}_1/2),\\&\mathbf{k}_3 = \mathbf{F}(t_n + h/2, \mathbf{y}_n + h \mathbf{k}_2/2), \quad \mathbf{k}_4 = \mathbf{F}(t_n + h, \mathbf{y}_n + h \mathbf{k}_3). \end{aligned}$$

Given a dataset $(\mathbf{y}(t))$, using the Levenberg–Marquardt algorithm, we aim to find the parameters $(\beta _n,\mu _n):=(\beta (t_n), \mu (t_n))$ of the model curve with the least-squares curve-fitting^45^,

5 $$\begin{aligned} ({{\hat{\beta }}}, {{\hat{\mu }}}) = {{\,\mathrm{arg\,min}\,}}_{\beta , \mu } \sum _{i=1}^m \left[ \mathbf{y}_{n+1,i} - \left( \mathbf{y}_{n,i} + \dfrac{h}{6} \left( \mathbf{k}_1 + 2 \mathbf{k}_2 + 2 \mathbf{k}_3 + \mathbf{k}_4 \right) \right) \right] ^2. \end{aligned}$$

We note that the Covid-19 dataset for each country is obtained from the Google mobility report^32^.

### Neural network architecture

Long short term memory networks—so-called LSTM—are variants of recurrent neural network (RNN), capable of learning long-term dependencies. They were introduced by Hochreiter and Schmidhuber^46^, and are widely used in many fields such as time series prediction^47^, speech recognition^48^, and robot control^49^ among many other applications.

Classic RNNs can keep track of arbitrary long-term dependencies in the input sequences. However, there is a computational drawback to the standard RNNs. In standard RNNs, this repeating module will have a very simple structure, such as a single layer. When training a classical RNN with back-propagation, the gradients which are back-propagated may tend to zero (vanish gradient problem) because the RNN remembers data for just a small duration of time. In other words, if we need the information after a small-time it may be reproducible, but once a lot of information is fed in, this information may get lost somewhere. This issue can be resolved by applying a variant of RNNs such as the LSTM network. The LSTMs are explicitly designed to avoid the long-term dependency problem as remembering information for long periods is practically their default behavior.

The compact forms of the LSTM with a forget gate can be described by the following system of equations:

6 $$\begin{aligned} f_t&= \sigma _g(W_{f} x_t + U_{f} h_{t-1} + b_f), \end{aligned}$$

7 $$\begin{aligned} i_t&= \sigma _g(W_{i} x_t + U_{i} h_{t-1} + b_i), \end{aligned}$$

8 $$\begin{aligned} o_t&= \sigma _g(W_{o} x_t + U_{o} h_{t-1} + b_o), \end{aligned}$$

9 $$\begin{aligned} \tilde{c}_t&= \sigma _c(W_{c} x_t + U_{c} h_{t-1} + b_c), \end{aligned}$$

10 $$\begin{aligned} c_t&= f_t \circ c_{t-1} + i_t \circ \tilde{c}_t, \end{aligned}$$

11 $$\begin{aligned} h_t&= o_t \circ \sigma _h(c_t), \end{aligned}$$

where $x_t$ is input vector, $c_t$ is a memory cell, and $\{i_t,f_t,o_t\}$ denote the input, forget, and output gates, respectively; for more details, see for instance^46,50,51^. Here, the operator $\circ$ denotes the Hadamard product (element-wise product), and subscript t indexes the time step.

**Figure 9:**
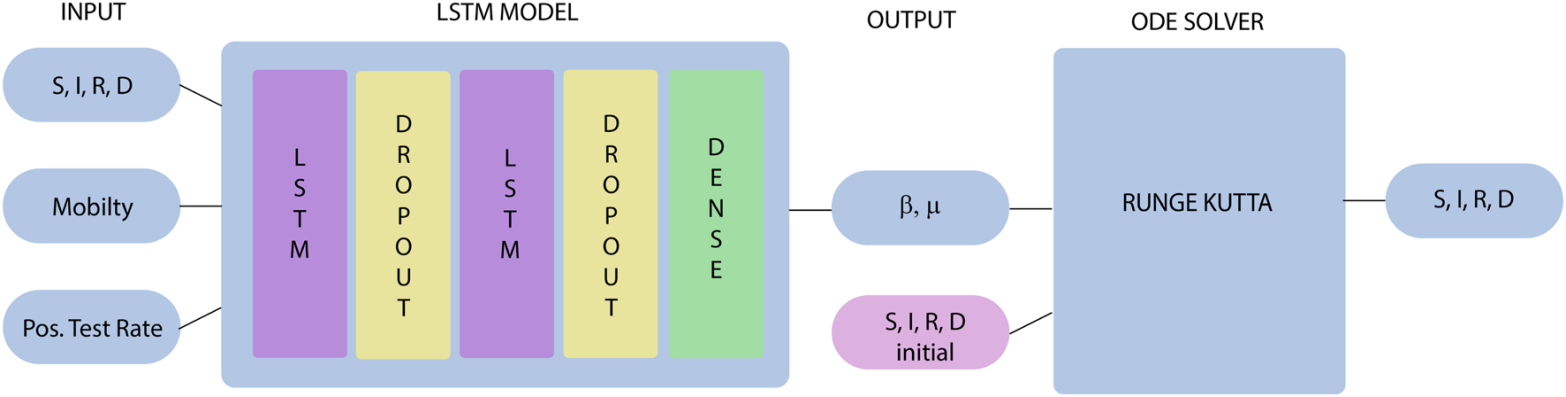
A description of the combined SIRD–LSTM model structure with Covid-19 community mobility (mobility) and positive test rate (Pos. Test Rate) to generate forecasts of time varying parameters $(\beta ,\mu )$. The ODE solver based on the Runge–Kutta fourth order method makes use of the predicted parameters in the numerical discretization.

In the proposed neural network, we couple the SIRD model (2) and the LSTM network. By the Levenberg–Marquardt algorithm, predictions on $\beta$ and $\mu$ are made by curve-fitting methods. With this, input data consists of $x_t = \{S_t, I_t, R_t, D_t, p_t, m_t, \beta _t, \mu _t\}$ where $p_t$ is a positive rate (the percentage of all coronavirus tests performed that are actually positive) and $m_t$ is a mobility trend at time *t* obtained from Google’s mobility report. The reports chart movement trends over time by geography, across different categories of places such as retail and recreation, groceries and pharmacies, parks, transit stations, workplaces, and residential. The parameters $\beta _t$ and $\mu _t$ are predicted by the Levenberg-Marquardt algorithm. The output of the LSTM network is ($\beta _{t+1}$, $\mu _{t+1})$. When implementing cost functions, we apply a mean-squared forecasting error metric as well as mean-absolute percentage errors.

The network structure and activation of each hidden unit in the hidden layers are determined by the neurons in the previous layers. The activity of each layer is given by the nonlinear activation function $\sigma$ such as a sigmoid function or ReLU function. The final output of the coupled model is obtained by combining the network output of confirmed cases with the SIR model forecast. More precisely, the collective dataset generated from the SIRD model is used as inputs for the LSTM whose outputs provide the parameters $\beta$ and $\mu$ for the next time period. By predicting the parameters, we are able to solve the SIRD moded, which gives $\{S, I, R, D\}$ for the next time period. The coupled models given in Fig. 9 illustrate the Neural LSTM-SIRD architecture. The network architecture we use is an LSTM with ReLU activation functions, and is trained by using Adam optimizer with a mean-squared error loss function. The model is not constrained to a particular setup and we could search over various hyperparameters to manipulate the number of neurons, with similar results.

### Data

We collected data from the following sources:

- Covid-19 data repository by the center for systems science and engineering (csse) at Johns Hopkins University, https://github.com/CSSEGISandData/COVID-19 (see^52^).
- Our World In Data, https://github.com/owid/covid-19-data/tree/master/public/data (see^53^).
- Google Mobility Report, https://www.google.com/covid19/mobility/.
